# Supplementary material for: What are the effects of implementing patient-controlled admissions in inpatient care? A study protocol of a large-scale implementation and naturalistic evaluation for adult and adolescent patients with severe psychiatric conditions throughout Region Stockholm
Source: BMJ Open. 2022 Aug 16;12(8):e065770. doi: 10.1136/bmjopen-2022-065770 (PMC9386218; doi:10.1136/bmjopen-2022-065770)
Supplement: Author's manuscript [file bmjopen-2022-065770.draft_revisions.pdf]

BMJ Open

BMJ Open is committed to open peer review. As part of this commitment we make the peer review history of every article we publish publicly available.

When an article is published we post the peer reviewers' comments and the authors' responses online. We also post the versions of the paper that were used during peer review. These are the versions that the peer review comments apply to.

The versions of the paper that follow are the versions that were submitted during the peer review process. They are not the versions of record or the final published versions. They should not be cited or distributed as the published version of this manuscript.

BMJ Open is an open access journal and the full, final, typeset and author-corrected version of record of the manuscript is available on our site with no access controls, subscription charges or pay-per-view fees (http://bmjopen.bmj.com).

If you have any questions on BMJ Open's open peer review process please email info.bmjopen@bmj.com

BMJ Open

Patient-controlled admissions to inpatient care: A study protocol of a large-scale implementation and naturalistic evaluation for adult and adolescent patients with severe psychiatric conditions in Region Stockholm

Journal:	*BMJ Open*
Manuscript ID	bmjopen-2022-065770
Article Type:	Protocol
Date Submitted by the Author:	16-Jun-2022

Complete List of Authors:	Smitmanis Lyle, Maria; Stockholm Health Care Services, Department of Clinical Neuroscience<br>Allenius, Emelie; Stockholm Health Care Services, Department of Clinical Neuroscience<br>Salomonsson, Sigrid; Stockholm Health Care Services, Department of Clinical Neuroscience<br>Björkdahl, Anna; Stockholm Health Care Services, Department of Clinical Neuroscience<br>Strand, Mattias; Stockholm Health Care Services, Department of Clinical Neuroscience<br>Flykt, L; Karolinska Institutet<br>Hellner, Clara; Karolinska Institute, Clinical neuroscience<br>Lundgren, Tobias; Karolinska Institute, Clinical Neuro Science ;<br>Jayaram-Lindström, Nitya; Karolinska Institutet, Centre for Psychiatry Research, Department of Clinical Neuroscience<br>Rozental, Alexander; Stockholm Health Care Services, Department of Clinical Neuroscience; Uppsala University, Department of Psychology

Keywords:	Adult psychiatry < PSYCHIATRY, Child & adolescent psychiatry < PSYCHIATRY, PSYCHIATRY

SCHOLARONE™
Manuscripts

Patient-controlled admissions to inpatient care: A study protocol of a large-scale implementation and naturalistic evaluation for adult and adolescent patients with severe psychiatric conditions in Region Stockholm

Maria Smitmanis Lyle[1]; Emelie Allenius[1]; Sigrid Salomonsson[1]; Anna Björkdahl[1]; Mattias Strand[1]; Lena Flyckt[1]; Clara Hellner[1]; Tobias Lundgren[1]; Nitya Jayaram-Lindström[1]; Alexander Rozental[1,2*]

1 Centre for Psychiatry Research, Department of Clinical Neuroscience, Karolinska Institutet, Stockholm County Council, Stockholm Health Care Services, Stockholm
2 Department of Psychology, Uppsala University

* Corresponding author: Alexander Rozental, PhD, Centre for Psychiatry Research, Department of Clinical Neuroscience, Karolinska Institutet, 117 63 Stockholm
Phone: +46 73 693 79 48, Email: alexander.rozental@ki.se

For peer review only

Abstract

Introduction: Patient-Controlled Admissions (PCA) represent a change in psychiatric inpatient care where patients are allowed to decide for themselves when hospitalization might be required. Prior research has demonstrated that PCA increase the number of admissions, but decrease days in inpatient care, while both the admissions to and days in involuntary care decrease. However, investigations have been restricted to specific patient groups and have not examined other possible benefits, such as effects on symptoms, quality of life, and autonomy. **~~Design and methods~~Methods and analysis:** The present study explores the implementation process and effects of PCA in Region Stockholm, who is currently introducing PCA for all patients with severe psychiatric conditions and extensive healthcare utilization. In total, the study comprises approximately 45 inpatient wards, including child and adolescent psychiatry. In a naturalistic evaluation, patients assigned PCA will be followed up to 36 months, both with regard to hospitalization rates and self-reported outcomes. In addition, qualitative studies will explore the experiences of patients, caregivers of adolescents, and healthcare providers. **Ethics and dissemination:** Approval has been granted by the Swedish Ethical Review Authority (Dnr: 2020-06498). Registration as a clinical trial has been made on www.ClinicalTrials.gov (NCT04862897). The findings from the present study will be disseminated via publications in international peer-reviewed journals, at scientific conferences, as part of two doctoral theses, and through the Swedish Partnership for Mental Health (NSPH).

Article summary

- Patient-Controlled Admissions (PCA) allow patients with severe psychiatric conditions to decide for themselves when inpatient care is warranted.

- The present study will examine hospitalization rates and self-reported outcomes for patients assigned PCA in Region Stockholm, Sweden.

- Patient with different psychiatric disorders from approximately 45 inpatient wards with will be included and followed up to 36 months.

- For the first time, the implications of PCA for child and adolescent psychiatry will be investigated.

- Qualitative studies will explore the experiences of patients, caregivers of adolescents, and healthcare providers.

For peer review only

Introduction

Patients with severe psychiatric conditions, such as schizophrenia spectrum disorders, borderline personality disorder, and eating disorders, often have care needs that warrant frequent admissions to inpatient care. Historically, it has been up to the healthcare providers to decide who and when to admit, sometimes referred to as the gate-keeper model. In essence, patients may seek care on their own, but the final decision to permit admission has always been made by a physician.[1] However, recent trends toward patient participation and shared decision-making in healthcare has questioned this practice.[2] In particular, the concept of Patient-Controlled Admissions (PCA) marks a dramatic shift in authority and increased partnership in psychiatric inpatient care, whereby patients are given the possibility of admitting themselves when needed. The basic premise is to identify and grant PCA to those who might benefit the most from its use as a way of preventing deterioration and novel bouts of illness and support patient empowerment. The procedures differ somewhat between settings and patient groups, but typically involves three to five inpatient days per stay, with or without restrictions regarding the number of admissions allowed per month.[1]

The effects of PCA have been examined in several studies. Early attempts of implementation were done in the United States and Australia, but the first large-scale investigations were made in the Netherlands, Norway, Denmark, and Sweden.[3-10] In sum, research indicates an increase in the number of admissions to inpatient care, but a decrease in the number of days in inpatient care. Meanwhile, both the admissions to and days in involuntary care decrease, suggesting that patients admit themselves more often, but require less fewer admissions of shorter duration, in line with the idea of using PCA to promote independence and increase help-seeking behaviors. However, apart from hospitalization rates, self-rated outcomes have been far less explored. Aspects such as recovery and well-being do not demonstrate any significant changes, but self-report measures have been few and mainly focused on general functioning.[4 8 9] Yet, qualitative studies imply that PCA can be beneficial in terms of increasing autonomy, agency, and well-being among patients. This is evident even in those cases where admissions are seldom or never used, suggesting that it might constitute a safety net for those at risk.[11]

PCA has been available since 2014 in Region Stockholm's psychiatric care, which is Sweden's largest hospital sector, but only for specific patient groups; schizophrenia spectrum disorders,[7] eating disorders,[9] and borderline personality disorder.[12] Different procedures for using PCA have also been used depending on diagnosis, such as the length of each stay. However, following a resolution by the popularly elected Region Stockholm Assembly, the implementation of PCA will be completed in 2023 and involve patients with severe psychiatric conditions, irrespective of diagnosis, using the same procedures in the whole region. This will allow a large-scale implementation and evaluation of its effects, both in terms of hospitalization rates and self-reported outcomes, up to 36 months following the assignment to PCA. A large number of patients with many different diagnoses will take part in several quantitative studies; including substance use, which has never been the subject to evaluation of PCA before. Furthermore, the use of additional self-report measures will permit an examination of its impact on symptoms, quality of life, and autonomy. In addition, the implementation of PCA in child and adolescent psychiatry will be the first of its kind, making it possible to examine its potential effects among adolescents. Lastly, qualitative studies will examine the experiences of patients, caregivers of adolescents, and healthcare providers in an attempt to understand the use, and potential benefits and drawbacks of PCA. Overall, approximately 45 inpatient wards will eventually be included in the present study, thus being, to the best of the authors' knowledge, the largest attempt at defining the effects of PCA.

For peer review only - http://bmjopen.bmj.com/site/about/guidelines.xhtml

Aims and objectives

The overall aim of the present study is to assess the effects of implementing PCA throughout Region Stockholm, using a naturalistic prospective study design. This involves a number of quantitative studies which intend to understand the impact of PCA on hospitalization rates and self-reported outcomes when introduced in psychiatric care. Qualitative studies will also be conducted to explore the experiences of both patients and healthcare providers with regard to its effects and use, including the perspective of caregivers of adolescents.

Primary research question:

For patients with severe psychiatric conditions, identified as having extensive care needs and who have been assigned PCA by their healthcare providers; how will their hospitalization rates change during the following 12, 24, and 36 months with regard to the number of admissions and days in inpatient care, and the number of admissions and days in involuntary care?

Secondary research question:

1) Will hospitalization rates differ between the various patient groups who have been assigned PCA?

2) Will self-reported outcomes change following the assignment of PCA, i.e., symptoms, quality of life, and autonomy?

3) Will self-reported outcomes differ between the various patient groups who have been assigned PCA?

4) How are the health care expenditures for patients who have been assigned PCA affected, and do they differ between the various patient groups?

5) What are the experiences of patients who have been assigned PCA for at least 12 months?

6) What are the experiences of healthcare providers who have worked with PCA for at least 12 months?

7) For adolescents, what are the experiences of caregivers to patients who have been assigned PCA for at least 12 months?

8) For adolescents, will self-reported outcomes change in terms of their caregivers' perception of the patients' symptoms and quality of life?

In line with prior research, the present study hypothesizes that a) the hospitalization rates of patients will increase in terms of number of admissions to inpatient care, but b) decrease in terms of days in inpatient care, as well as number of admissions and days in involuntary care. Furthermore, self-reported outcomes are expected to demonstrate c) a decrease in symptoms, and d) increase in quality of life and autonomy. Lastly, qualitative investigations are believed to give a greater understanding of how patients, caregivers of adolescents, and healthcare providers experience the use of PCA.

Method and analysis

Setting

For peer review only

The present study is placed within the psychiatric care of Region Stockholm, which is the largest hospital sector in Sweden, serving a population of two million citizens. It is organized as nine public healthcare clinics, and four private healthcare clinics. One of these clinics constitute child and adolescent psychiatry, two concern eating disorders, and two are dedicated to substance use disorders. One additional clinic is reserved for forensic psychiatry, but because its patients have been sentenced to care, these are not eligible to receive PCA. The 13 clinics that are included consist of approximately 45 inpatient wards and about 120 outpatient units that work together as pairs (i.e., more than one outpatient unit can share the same inpatient ward). Each inpatient ward will in turn designate at least one bed for PCA. The healthcare providers consist of medical doctors and nurses with or without specialist training in psychiatry, mental health workers, counsellors, psychologists, psychotherapists, social workers, physiotherapists, occupational therapists, and students undergoing basic or advanced clinical training in the field of medicine or psychiatry.

Patients

In Region Stockholm, PCA is assigned to those patients that have a severe psychiatric condition and who are deemed by their healthcare providers to warrant great care needs. Thus, eligible patients present with a documented history of recurrent admissions to inpatient care, a large number of days in inpatient care, or other indications of future hospitalization. In order to determine eligibility, the following criteria are used by the healthcare providers:

- Ongoing contact with an outpatient unit.
- An individual care plan and crisis plan.
- At least one episode of inpatient care during the previous one-year period.
- Expected future need of inpatient care.
- Demonstrating an interest in and understanding the concept of PCA (for adolescents, this also includes their caregivers).

The inpatient wards include patients from general psychiatry (e.g., schizophrenia spectrum diagnoses, neuropsychiatric disorders, and anxiety disorders), geriatric psychiatry, affective disorders (e.g., bipolar disorder), substance use disorders, personality disorders, eating disorders, and child and adolescent psychiatry (i.e., up to 18 years of age).

Procedure

Patients who are eligible to receive PCA are invited to a scheduled meeting with their healthcare providers from the outpatient unit and inpatient ward. The purpose and practice of PCA are then discussed verbally and information is provided in writing. Should the patient accept the offer, an agreement on the use of PCA is reached collaboratively and recorded in their medical records. For adolescents, caregivers also have to agree. Typically, PCA involves up to four days of inpatient care at a time, up to three times per month, although exceptions to the rule can sometimes be made. The agreement only covers 12 months at a time, after which it can be renewed at the yearly assessments. After the meeting, the patient receives all of the details in text and a printed copy of the agreement.

Each inpatient ward has allocated at least one inpatient bed for patients assigned PCA. Should a patient with an agreement wish to be admitted, they only have to contact the inpatient ward, any time of the day, any day of the week. In case the inpatient bed is already occupied, the patient is put on a waiting list. If more immediate care is required, the patient can always be admitted as usual. All admissions are managed by a nurse with delegated responsibility from the head of clinic. During admission, the patient is assessed with regard to

For peer review only

suicidality, violence, and vital parameters, such as blood pressure and body temperature. Should there be an increased risk (e.g., suicidal ideation and physical injury), regular intake is made. Once admitted, patients are offered care according to their specific needs and individual care plan. Consultation with a medical doctor is possible, but not part of usual practice. The patient is also free to discharge at will, which is also handled by a nurse; however, should the patient warrant further stay at the inpatient ward, a medical doctor can decide if regular admittance is necessary.

Since PCA is part of regular healthcare, patients can always agree to its use but refuse to participate in the present research study. The offer to take part in a study on the effects of PCA is always made after the use of PCA first has been agreed upon, whereupon the objectives of the present study is discussed verbally and information is provided in writing. Informed consent is required and documented in the medical records. For adolescents, informed consent needs to be signed by both the adolescent and all caregivers (e.g., legal parents, custodians, or other entities responsible for the patient's care). Following this, the patient completes the self-reported outcome questionnaires that are included in the study, as reviewed below. For adolescents, this also involves the caregivers' perception of the patient's well-being. All of the self-reported outcome questionnaires are then completed at the mandatory yearly assessments, except for the Visual Analogue Scales (VAS) that are administered each month via SMS (more information provided under self-reported outcomes). This is in line with the procedures surrounding the use of PCA in Region Stockholm, where mandatory yearly assessments are made to check up on patients' health status and whether or not PCA should be continued. This is also believed to improve data collection as it does not require paper-and-pencil administration or letters.

The implementation of PCA throughout Region Stockholm is coordinated and supported by the Centre for Psychiatry Research, which is a R&D center run jointly by Karolinska Institutet and the hospital sector. The Centre for Psychiatry Research performs an assessment of the risks and needs of each outpatient unit and inpatient ward, and provides training and supervision regarding all of the PCA routines. This consists of an online course and a half-day on-site workshop, and the latter of regular meetings with all of the healthcare providers. The Centre for Psychiatry Research is also responsible for evaluating the effects of PCA and has a research steering group consisting of the authors of the current study protocol. These range from doctoral candidates to senior-level researchers with comprehensive experience of conducting both quantitative and qualitative studies in a psychiatric setting.

Study design

The present study will evaluate the effects of PCA in Region Stockholm in collaboration with the Commissioner of Healthcare. This follows a resolution made by the popularly elected Region Stockholm Assembly, which means that both the implementation and policy surrounding PCA have already been decided. Hence, according to the decision, all patients with severe psychiatric conditions who are considered by their healthcare providers to have great care needs will be offered PCA as long as the criteria mentioned above are fulfilled. Furthermore, because the primary objective of the PCA program is the provision of healthcare and not research, a more complex study design using randomization on an individual or clinic level (i.e., cluster) is not allowed due to ethical and judicial reasons. Hence, the present study is restricted to a naturalistic prospective study design. Patients are followed for 12, 24, and 36 months using their medical records to check changes in hospitalization prior to (pre) and following inclusion in the PCA program (post), similar to what has been done previously.[7] For example, patients who are included in November 2021

For peer review only

may have hospitalization rates retrieved from the period November 2018-2020 and compared against hospitalization rates from the period November 2022-2024, depending on the availability of historical data. In terms of self-reported outcomes, patients complete the measures when being assigned PCA and at the yearly follow-up assessments, with the exception for the VAS that is administered monthly via SMS. However, in these specific cases, historical data do not exist as these measures have not been collected previously. Only patients with no previous use of PCA when they consent to take part in the present study will be analyzed, as recorded in their hospital records.

Demographic variables
Demographic variables are recorded in the patients' medical records through their social security number. These are age, gender, civil status, and children. Psychiatric disorders are entered manually by healthcare providers using the Swedish version of the International Classification of Diseases and Related Health Problems, 10th Revision (ICD-10-SE).

Hospitalization rates
Hospitalization rates are recorded in the patients' medical records when admitted to inpatient care or involuntary care (i.e., when and how long each stay lasted). This consists of number of admissions and days in inpatient care, and number of admissions and days in involuntary care. All of the healthcare providers have been trained in how to enter this information in the system, including coding schemes specifically designed for the present study in order to identify and track patients.

Self-reported outcomes
Self-reported outcome (i.e., questionnaires) are collected after the patients have been assigned PCA and consented to take part in the research. For an overview, see Table 1.

Table 1
Overview of The Self-Reported Outcomes

	Assessment				
**Self-report measure**	**Assigned PCA**	**Monthly measures**	**12 months**	**24 months**	**36 months**
**Adults**					
EQ-5D-5L	X		X	X	X
WHODAS	X		X	X	X
CGI[a]	X		X	X	X
BBQ	X		X	X	X
GSE	X		X	X	X
GAD-7	X		X	X	X
PHQ-9	X		X	X	X
VAS	X	X	X	X	X
**Adolescents**					
KIDSCREEN	X		X	X	X
ASSIST-Y	X		X	X	X
C-GAS[a]	X		X	X	X
SDQ[b]	X		X	X	X
RCADS[b]	X		X	X	X

WHODAS = World Health Organization Disability Assessment Schedule 2.0; CGI = Clinical Global Impression; BBQ = Brunnsviken Brief Quality of Life Scale; GSE = General Self-Efficacy Scale; GAD-7 =

For peer review only

Generalized Anxiety Disorder – 7 Items; PHQ-9 = Patient Health Questionnaire – 9 Items; VAS = Visual Analogue Scales; ASSIST-Y = Alcohol, Smoking and Substance Involvement Screening Test; C-GAS = Children's Global Assessment Scale; SDQ = The Strengths and Difficulties Questionnaire; RCADS = Revised Children's Anxiety and Depression Scale
[a] Clinician-rating scale
[b] Also completed by caregivers

EQ-5D-5L: A five-item questionnaire on current health status on the dimensions; mobility, self-care, usual activities (e.g., family), pain/discomfort, and anxiety/depression. Each item is scored in relation to a five-step scale, e.g., "I am not anxious or depressed" (1) to "I am extremely anxious or depressed" (5). Furthermore, EQ-5D-5L also includes a VAS on current health status; "Worst imaginable health" (0) to "Best imaginable health" (100).[13]

World Health Organization Disability Assessment Schedule 2.0 (WHODAS): A 12-item questionnaire on overall physical and psychological well-being, e.g., "In the past 30 days, how much difficulty did you have in: Taking care of household responsibilities?". It is scored on five-point Likert-scale, "None" (1) to "Extreme or cannot do" (5). Three additional items also concern the number of days difficulties were present or interfered with daily living.[14]

Clinical Global Impression (CGI): A clinician-rating scale that asks healthcare providers to rate the health status of a patient at an initial assessment (referred to CGI-S); "Normal, not at all ill" (1) to "Among the most extremely ill patients" (7). The CGI is also used to determine the improvement of a patient (referred to as CGI-I); "Very much improved since the initiation of treatment" (1) to "Very much worse since the initiation of treatment" (7).[15]

Brunnsviken Brief Quality of Life Scale (BBQ): A 12-item questionnaire on the quality of life in six domains; leisure time, view of life, creativity, learning, friends and friendship, and myself as a person. Each domain is scored in accordance with satisfaction and importance, "Do not agree at all" (0) and "Agree completely" (4), which are then multiplied. The sum of the six products constitutes the total quality of life score.[16]

General Self-Efficacy Scale (GSE): A 10-item questionnaire on self-efficacy, i.e., the belief to succeed in a given situation or task, e.g., "I can always manage to solve difficult problems if I try hard enough" (Item 1). It is scored on a four-point Likert-scale, "Not at all true" (1) to "Exactly true" (4).[17]

Generalized Anxiety Disorder – 7 Items (GAD-7): A seven-item questionnaire on symptoms of anxiety and worry, e.g., "How often have they been bothered by the following over the past 2 weeks? Feeling nervous, anxious, or on the edge" (Item 1). It is scored on a four-point Likert-scale, "Not at all" (0) to "Nearly every day" (3).[18]

Patient Health Questionnaire – 9 Items (PHQ-9): A nine-item questionnaire on symptoms of depression and mood, e.g., "How often have they been bothered by the following over the past 2 weeks? Little interest or pleasure in doing things?" (Item 1). It is scored on a four-point Likert-scale, "Not at all" (0) to "Nearly every day" (3).[19]

Visual Analogue Scales (VAS): Four items assessing different aspects of the patients' care needs and current health status are administered monthly via SMS. These include the following; 1) "I feel confident about receiving care when I need it" 2) "I am able to actively participate in my care", and 3) "I believe care is available when needed". These are scored on a continuum ranging from "I do not agree at all" (0) to "Totally agree" (10). Also, a fourth

For peer review only

item concerns “How would you rate your current health status?”, which is rated between “Worst possible health” (0) and “Best possible health” (10).

For adolescents and their caregivers, a separate set of self-reported outcomes will be used.

KIDSCREEN: An 11-item questionnaire on aspects related to health-related quality of life, e.g., “Thinking about the last week: have you been able to do the things that you want to do in your free time?” (Item 1). It is scored on a five-point Likert-scale, “Not at all” or “Never” (0) to “Extreme” or “Always” (4). One item is supposed to reflect the participant’s overall health status, i.e., “In general, how would you say your health is?”. KIDSCREEN is administered to both the patients and their caregivers.[20]

Alcohol, Smoking and Substance Involvement Screening Test (ASSIST-Y): A seven-item questionnaire on alcohol, substance, and tobacco use among adolescents. It screens for the consumption and negative effects of different compounds, similar to AUDIT and DUDIT, weighting scores differently depending on frequency and impact of their use.[21]

Children’s Global Assessment Scale (C-GAS): A clinician-rating scale that asks healthcare providers to rate the health status of a patient. It is scored on a continuum ranging from “Needs constant supervision” (1-10) to “Superior functioning” (100).[22]

The Strengths and Difficulties Questionnaire (SDQ): A 25-item questionnaire on the psychological well-being of an adolescent, e.g., “Often loses temper” (Item 5). It is scored on a three-point Likert-scale, “Not true” (0) to “Certainly true” (2). SDQ is administered to both the patients and their caregivers.[23]

Revised Children's Anxiety and Depression Scale (RCADS): A 47-item questionnaire covering different symptoms of different psychiatric conditions, e.g., “I worry that something bad will happen to me” (Item 27). It is scored on a four-point Likert-scale, “Never” (0) to “Always” (3). RCADS is administered to both the patients and their caregivers.[24]

A separate study will also investigate how the use of PCA among adolescents affect their caregivers’ quality of life (BBQ), functioning (Burden Assessment Scale),[25] and the perceived reception by healthcare providers (Family Involvement and Alienation Questionnaire).[26]

Sample size calculation

The present study intends to examine the impact of introducing PCA in psychiatric care in Region Stockholm. As such, only patients who are assigned PCA and have consented to take part in the research are included. An a priori sample size calculation, which is typically used to estimate an adequate sample size for subsequent recruitment, is thus not informative. However, based on previous experiences of introducing PCA in the region, it is reasonable to expect about twelve patients per inpatient ward who consent to participate, which would amount to a total sample size of 564.[7] When calculating the standardized effect size possible to detect under these circumstances, based on the difference between two dependent means, and using a probability level of .05 and a power of 0.80, the result is Cohen’s d of 0.12, which is a small, albeit clinically relevant effect for a population of patients with severe psychiatric disorders, and in line with similar findings on self-reported outcomes.[4 8 9]

Statistical analysis

For peer review only

Previous studies on the use of PCA have demonstrated that hospitalization rates are seldom normally distributed. In essence, few patients will use the possibility to admit themselves to inpatient care, resulting in data that is positively skewed and has a leptokurtic distribution. Investigating whether assumptions of normality are violated will therefore be essential prior to any statistical analysis, whereby suitable means of analyzing the data will be employed, that is, non-parametric tests or bootstrapping procedures. As for the self-reported outcomes, no prior evidence seems to suggest that data will not be normally distributed. Here, conditional changes over time will instead be modeled using a mixed models-approach, i.e., multilevel models. All analyses will be made according to an intent-to-treat principle, accounting for those values that have been lost via multiple imputation or maximum likelihood estimation. Likewise, all analyses will control for potential confounders, such as hospitalization rates prior to PCA and self-reported outcomes when assigned PCA, as well as demographics available in the hospital records, e.g., age, gender, civil status, children, and psychiatric disorders.

Apart from studying the results of introducing PCA, the present study will also assess its cost-effectiveness, similar to a newly published study from Denmark.[10] This includes determining the direct and total healthcare costs prior to and after being assigned PCA. Direct costs are the expenditures associated with each visit to a psychiatric emergency department, inpatient care, and involuntary care. Such expenditures are calculated from standardized price estimates provided by Region Stockholm, resulting in an approximation of how expenditures change following the implementation of PCA. Total healthcare costs are the expenditures of other types of healthcare, e.g., visits to primary care. Moreover, cost-~~effectiveness~~ utility analyses will also entail so-called Quality Adjusted Life Years (QALYs), which represent the improvement in terms of well-being for each incremental increase in costs by allowing patients to admit themselves, i.e., a cost-utility approach. QALYs can be derived from self-reported outcomes on the EQ-5D-5L, which is commonly used for this purpose, using population-generated preference weights which are available for a Swedish setting.[27] This will be compared to matched patients using registries. In addition, probability sensitivity analyses will be conducted to determine the errors that surrounds the cost-~~effectiveness~~ utility calculations made. This provides confidence ellipses at 50%, 75% and 95% that represent the uncertainty associated with each estimate.

Qualitative studies

In addition to the quantitative studies, the present study will also involve a number of qualitative investigations to further the current understanding of its use and effects, similar to previous research in the field[28 29]. First, patients who have been assigned PCA for at least 12 months will be recruited to share their perspective (n = 30). Recruitment will be purposeful to ensure heterogeneity with regard to patient groups and the extent to which PCA have been used. The aim is to explore how PCA has been perceived and what impact it might have had on both day-to-day activities and quality of life. Second, healthcare providers from different inpatient wards and professional backgrounds, having had at least 12 months of experience of working with PCA, will be recruited to provide their view of its application (n = 30). The aim is to investigate their attitudes toward the method in general, the implementation of PCA in particular, and whether these have changed over time as a result of working with PCA. Third, caregivers of adolescents who have been assigned PCA for at least 12 months will be recruited to share their experiences (n = 20), both in terms of how they have perceived its effects for the adolescent and how it might have affected their own situation. All of the qualitative studies will be made using individual semi-structured interviews that are recorded digitally and transcribed ad verbum. Given the exploratory nature of this research, all

For peer review only

analyses will be inductive and done in accordance with thematic analysis.[30] This approach is often used in social sciences as well as in health sciences and helps to understand the viewpoint of an individual in relation to a specific topic, phenomenon, or subject matter. This can be particularly useful in examining patterns and concepts in shared responses, which is helpful in generating hypotheses in a research field that lacks prior research.

Ethics and dissemination

The present study was approved in March 2021 by the Swedish Ethical Review Authority (Dnr: 2020-06498), and has been registered as a clinical trial on www.ClinicalTrials.gov (NCT04862897). All data is recorded using Region Stockholm's public healthcare's digital medical records system, TakeCare, and only exported for statistical analyses in an aggregated pseudonymized format. Moreover, participant identifiers instead of Swedish social security numbers are used to ensure anonymity, e.g., LSR64UFL. To participate in the research, informed consent has to be provided, and for adolescents, this includes caregivers (e.g., legal parents, custodians, or other entities responsible for the patient's care).

Given the novelty of the intervention and lack of research concerning PCA, the present study has the potential to make a significant contribution to the field. Apart from examining its effects with regard to hospitalization rates, self-reported outcomes will help to investigate possible benefits on symptoms, quality of life, and autonomy. In addition, qualitative studies will provide useful insights regarding its use and implications for care and everyday life, including the opinions of caregivers of adolescents. In order to successfully disseminate the results, findings will be presented in international peer-reviewed journals, at scientific conferences, and as part of two doctoral theses. In addition, an ongoing collaboration with patient representatives will ensure Patient Public Involvement and dissemination to patients and their significant others, primarily through the Swedish Partnership for Mental Health (NSPH).

Author's contributions

AR, SS, TL, NJL, MSL, EA, AB, CH, MS and LF have all been involved in the design and planning of the present study. All authors have taken part in drafting the manuscript and are also part of the steering group for researching PCA in the region.

Funding statement

The present study, its implementation, and research is funded by the Commissioner of Healthcare in Region Stockholm.

Competing interest statement

None declared.

Patient and public involvement

The present study is conducted in collaboration with the Swedish Partnership for Mental Health (NSPH). Patient and public representatives are not involved in issues surrounding study design, but are informed about and actively engaged in formulating research questions, determining outcomes, and disseminating the results.

For peer review only

References

1. Strand M, von Hausswolff-Juhlin Y. Patient-controlled hospital admission in psychiatry: A systematic review. *Nord J Psychiatry* 2015;69(8):574-86. doi: 10.3109/08039488.2015.1025835 [published Online First: 2015/04/03]
2. Pelto-Piri V, Engström K, Engström I. Paternalism, autonomy and reciprocity: ethical perspectives in encounters with patients in psychiatric in-patient care. *BMC medical ethics* 2013;14(1):1-8.
3. Koekkoek B, van der Snoek R, Oosterwijk K, et al. Preventive psychiatric admission for patients with borderline personality disorder: a pilot study. *Perspect Psychiatr Care* 2010;46(2):127-34. doi: 10.1111/j.1744-6163.2010.00248.x [published Online First: 2010/04/10]
4. Moljord IEO, Lara-Cabrera ML, Salvesen O, et al. Twelve months effect of self-referral to inpatient treatment on patient activation, recovery, symptoms and functioning: A randomized controlled study. *Patient Educ Couns* 2017;100(6):1144-52. doi: 10.1016/j.pec.2017.01.008 [published Online First: 2017/01/18]
5. Moljord IE, Helland-Hansen KA, Salvesen O, et al. Short time effect of a self-referral to inpatient treatment for patients with severe mental disorders: a randomized controlled trial. *BMC Health Serv Res* 2016;16(1):513. doi: 10.1186/s12913-016-1712-z [published Online First: 2016/09/24]
6. Thomsen CT, Benros ME, Maltesen T, et al. Patient-controlled hospital admission for patients with severe mental disorders: a nationwide prospective multicentre study. *Acta Psychiatr Scand* 2018;137(4):355-63. doi: 10.1111/acps.12868 [published Online First: 2018/03/06]
7. Skott M, Durbeej N, Smitmanis-Lyle M, et al. Patient-controlled admissions to inpatient care: A twelve-month naturalistic study of patients with schizophrenia spectrum diagnoses and the effects on admissions to and days in inpatient care. *BMC Health Serv Res* 2021;21(1):598. doi: 10.1186/s12913-021-06617-8 [published Online First: 2021/06/25]
8. Westling S, Daukantaite D, Liljedahl SI, et al. Effect of Brief Admission to Hospital by Self-referral for Individuals Who Self-harm and Are at Risk of Suicide: A Randomized Clinical Trial. *JAMA Netw Open* 2019;2(6):e195463. doi: 10.1001/jamanetworkopen.2019.5463 [published Online First: 2019/06/08]
9. Strand M, Bulik CM, Gustafsson SA, et al. Self-admission to inpatient treatment in anorexia nervosa: Impact on healthcare utilization, eating disorder morbidity, and quality of life. *Int J Eat Disord* 2020 doi: 10.1002/eat.23346 [published Online First: 2020/07/16]
10. Paaske LS, Sopina L, Olsen KR, et al. The impact of patient-controlled hospital admissions among patients with severe mental disorders on health care cost: A nationwide register-based cohort study using quasi-experimental design. *J Psychiatr Res* 2021;144:331-37. doi: 10.1016/j.jpsychires.2021.10.032 [published Online First: 2021/11/06]
11. Olso TM, Gudde CB, Moljord IE, et al. More than just a bed: mental health service users' experiences of self-referral admission. *Int J Ment Health Syst* 2016;10:11. doi: 10.1186/s13033-016-0045-y [published Online First: 2016/02/27]
12. Eckerström J, Flyckt L, Carlborg A, et al. Brief admission for patients with emotional instability and self-harm: A qualitative analysis of patients' experiences during crisis. *International Journal of Mental Health Nursing* 2020 doi: 10.1111/inm.12736
13. Herdman M, Gudex C, Lloyd A, et al. Development and preliminary testing of the new five-level version of EQ-5D (EQ-5D-5L). *Quality of life research* 2011;20(10):1727-36.

For peer review only

14. Üstün TB, Kostanjsek N, Chatterji S, et al. Measuring health and disability: Manual for WHO disability assessment schedule WHODAS 2.0: World Health Organization 2010.
15. Busner J, Targum SD. The Clinical Global Impressions Scale: Applying a research tool in clinical practice. *Psychiatry (Edgmont)* 2007;4(7):28-37.
16. Lindner P, Frykheden O, Forsstrom D, et al. The Brunnsviken Brief Quality of Life Scale (BBQ): Development and Psychometric Evaluation. *Cogn Behav Ther* 2016;45(3):182-95. doi: 10.1080/16506073.2016.1143526 [published Online First: 2016/02/18]
17. Sherer M, Maddux JE, Mercandante B, et al. The self-efficacy scale: Construction and validation. *Psychological reports* 1982;51(2):663-71.
18. Spitzer RL, Kroenke K, Williams JB, et al. A brief measure for assessing generalized anxiety disorder: the GAD-7. *Archives of internal medicine* 2006;166(10):1092-97.
19. Kroenke K, Spitzer RL, Williams JB. The PHQ-9: validity of a brief depression severity measure. *Journal of general internal medicine* 2001;16(9):606-13.
20. Ravens-Sieberer U, Gosch A, Rajmil L, et al. KIDSCREEN-52 quality-of-life measure for children and adolescents. *Expert review of pharmacoeconomics & outcomes research* 2005;5(3):353-64.
21. Humeniuk R, Holmwood C, Beshara M, et al. ASSIST-Y V1. 0: First-Stage Development of the WHO Alcohol, Smoking and Substance Involvement Screening Test (ASSIST) and linked brief intervention for young people. *Journal of Child & Adolescent Substance Abuse* 2016;25(4):384-90.
22. Shaffer D, Gould MS, Brasic J, et al. A children's global assessment scale (CGAS). *Archives of General psychiatry* 1983;40(11):1228-31.
23. Goodman R. The Strengths and Difficulties Questionnaire: a research note. *Journal of child psychology and psychiatry* 1997;38(5):581-86.
24. Chorpita BF, Yim L, Moffitt C, et al. Assessment of symptoms of DSM-IV anxiety and depression in children: A revised child anxiety and depression scale. *Behaviour research and therapy* 2000;38(8):835-55.
25. Ewertzon M, Lützén K, Svensson E, et al. Developing the concept of family involvement and the alienation questionnaire in the context of psychiatric care. *International Journal of Mental Health Nursing* 2008;17(6):439-49.
26. Reinhard SC, Gubman GD, Horwitz AV, et al. Burden assessment scale for families of the seriously mentally ill. *Evaluation and program planning* 1994;17(3):261-69.
27. Burström K, Sun S, Gerdtham U-G, et al. Swedish experience-based value sets for EQ-5D health states. *Quality of Life Research* 2014;23(2):431-42.
28. Ellegaard T, Bliksted V, Lomborg K, et al. Use of patient-controlled psychiatric hospital admissions: patients' perspective. *Nord J Psychiatry* 2017;71(5):370-77. doi: 10.1080/08039488.2017.1302505 [published Online First: 2017/03/23]
29. Ellegaard T, Mehlsen M, Lomborg K, et al. Use of patient-controlled psychiatric hospital admissions: mental health professionals' perspective. *Nord J Psychiatry* 2017;71(5):362-69. doi: 10.1080/08039488.2017.1301548 [published Online First: 2017/03/21]
30. Braun V, Clarke V. Using thematic analysis in psychology. *Qualitative Research in Psychology* 2006;3(2):77-101. doi: 10.1191/1478088706qp063oa

For peer review only

BMJ Open

What are the effects of implementing patient-controlled admissions in inpatient care? A study protocol of a large-scale implementation and naturalistic evaluation for adult and adolescent patients with severe psychiatric conditions throughout Region Stockholm

Journal:	*BMJ Open*
Manuscript ID	bmjopen-2022-065770.R1
Article Type:	Protocol
Date Submitted by the Author:	02-Aug-2022
Complete List of Authors:	Smitmanis Lyle, Maria; Stockholm Health Care Services, Department of Clinical Neuroscience<br>Allenius, Emelie; Stockholm Health Care Services, Department of Clinical Neuroscience<br>Salomonsson, Sigrid; Stockholm Health Care Services, Department of Clinical Neuroscience<br>Björkdahl, Anna; Stockholm Health Care Services, Department of Clinical Neuroscience<br>Strand, Mattias; Stockholm Health Care Services, Department of Clinical Neuroscience<br>Flyckt, Lena; Karolinska Institutet<br>Hellner, Clara; Karolinska Institute, Clinical neuroscience<br>Lundgren, Tobias; Karolinska Institute, Clinical Neuro Science ;<br>Jayaram-Lindström, Nitya; Karolinska Institutet, Centre for Psychiatry Research, Department of Clinical Neuroscience<br>Rozental, Alexander; Stockholm Health Care Services, Department of Clinical Neuroscience; Uppsala University, Department of Psychology
**Primary Subject Heading**:	Mental health
Secondary Subject Heading:	Mental health, Patient-centred medicine, Public health
Keywords:	Adult psychiatry < PSYCHIATRY, Child & adolescent psychiatry < PSYCHIATRY, PSYCHIATRY

SCHOLARONE™
Manuscripts

What are the effects of implementing patient-controlled admissions in inpatient care? A study protocol of a large-scale implementation and naturalistic evaluation for adult and adolescent patients with severe psychiatric conditions throughout Region Stockholm

Maria Smitmanis Lyle[1]; Emelie Allenius[1]; Sigrid Salomonsson[1]; Anna Björkdahl[1]; Mattias Strand[1]; Lena Flyckt[1]; Clara Hellner[1]; Tobias Lundgren[1]; Nitya Jayaram-Lindström[1]; Alexander Rozental[1,2*]

1 Centre for Psychiatry Research, Department of Clinical Neuroscience, Karolinska Institutet, Stockholm County Council, Stockholm Health Care Services, Stockholm
2 Department of Psychology, Uppsala University

* Corresponding author: Alexander Rozental, PhD, Centre for Psychiatry Research, Department of Clinical Neuroscience, Karolinska Institutet, 117 63 Stockholm
Phone: +46 73 693 79 48, Email: alexander.rozental@ki.se

For peer review only

Abstract

Introduction: Patient-Controlled Admissions (PCA) represent a change in psychiatric inpatient care where patients are allowed to decide for themselves when hospitalization might be required. Prior research has demonstrated that PCA increase the number of admissions, but decrease days in inpatient care, while both the admissions to and days in involuntary care decrease. However, investigations have been restricted to specific patient groups and have not examined other possible benefits, such as effects on symptoms, quality of life, and autonomy. **Methods and analysis:** The present study explores the implementation process and effects of PCA in Region Stockholm, who is currently introducing PCA for all patients with severe psychiatric conditions and extensive healthcare utilization. In total, the study comprises approximately 45 inpatient wards, including child and adolescent psychiatry. In a naturalistic evaluation, patients assigned PCA will be followed up to 36 months, both with regard to hospitalization rates and self-reported outcomes. In addition, qualitative studies will explore the experiences of patients, caregivers of adolescents, and healthcare providers. **Ethics and dissemination:** Approval has been granted by the Swedish Ethical Review Authority (Dnr: 2020-06498). Registration as a clinical trial has been made on www.ClinicalTrials.gov (NCT04862897). The findings from the present study will be disseminated via publications in international peer-reviewed journals, at scientific conferences, as part of two doctoral theses, and through the Swedish Partnership for Mental Health (NSPH).

Strengths and limitations of this study

- The present study will examine hospitalization rates and self-reported outcomes for all patients assigned Patient-Controlled Admissions (PCA) in Region Stockholm, Sweden.

- Patient with different psychiatric disorders from approximately 45 inpatient wards with will be included and followed up to 36 months.

- For the first time, the implications of PCA for child and adolescent psychiatry will be investigated.

- Qualitative studies will explore the experiences of patients, caregivers of adolescents, and healthcare providers.

- Due to ethical and judicial reasons, the present study will not allow randomization of patients or clusters, affecting the possibility of drawing causal inferences of outcomes.

For peer review only

Introduction

Patients with severe psychiatric conditions, such as schizophrenia spectrum disorders, borderline personality disorder, and eating disorders, often have care needs that warrant frequent admissions to inpatient care. Historically, it has been up to the healthcare providers to decide who and when to admit, sometimes referred to as the gate-keeper model. In essence, patients may seek care on their own, but the final decision to permit admission has always been made by a physician.[1] However, recent trends toward patient participation and shared decision-making in healthcare has questioned this practice.[2] In particular, the concept of Patient-Controlled Admissions (PCA) marks a dramatic shift in authority and increased partnership in psychiatric inpatient care, whereby patients are given the possibility of admitting themselves when needed. The basic premise is to identify and grant PCA to those who might benefit the most from its use as a way of preventing deterioration and novel bouts of illness and support patient empowerment. The procedures differ somewhat between settings and patient groups, but typically involves three to five inpatient days per stay, with or without restrictions regarding the number of admissions allowed per month.[1]

The effects of PCA have been examined in several studies. Early attempts of implementation were done in the United States and Australia, but the first large-scale investigations were made in the Netherlands, Norway, Denmark, and Sweden.[3-10] In sum, research indicates an increase in the number of admissions to inpatient care, but a decrease in the number of days in inpatient care. Meanwhile, both the admissions to and days in involuntary care decrease, suggesting that patients admit themselves more often, but require less fewer admissions of shorter duration, in line with the idea of using PCA to promote independence and increase help-seeking behaviors. However, apart from hospitalization rates, self-rated outcomes have been far less explored. Aspects such as recovery and well-being do not demonstrate any significant changes, but self-report measures have been few and mainly focused on general functioning.[4 8 9] Yet, qualitative studies imply that PCA can be beneficial in terms of increasing autonomy, agency, and well-being among patients. This is evident even in those cases where admissions are seldom or never used, suggesting that it might constitute a safety net for those at risk.[11]

PCA has been available since 2014 in Region Stockholm's psychiatric care, which is Sweden's largest hospital sector, but only for specific patient groups; schizophrenia spectrum disorders,[7] eating disorders,[9] and borderline personality disorder.[12] Different procedures for using PCA have also been used depending on diagnosis, such as the length of each stay. However, following a resolution by the popularly elected Region Stockholm Assembly, the implementation of PCA will be completed in 2023 and involve patients with severe psychiatric conditions, irrespective of diagnosis, using the same procedures in the whole region. This will allow a large-scale implementation and evaluation of its effects, both in terms of hospitalization rates and self-reported outcomes, up to 36 months following the assignment to PCA. A large number of patients with many different diagnoses will take part in several quantitative studies; including substance use, which has never been the subject to evaluation of PCA before. Furthermore, the use of additional self-report measures will permit an examination of its impact on symptoms, quality of life, and autonomy. In addition, the implementation of PCA in child and adolescent psychiatry will be the first of its kind, making it possible to examine its potential effects among adolescents. Lastly, qualitative studies will examine the experiences of patients, caregivers of adolescents, and healthcare providers in an attempt to understand the use, and potential benefits and drawbacks of PCA. Overall, approximately 45 inpatient wards will eventually be included in the present study, thus being, to the best of the authors' knowledge, the largest attempt at defining the effects of PCA.

For peer review only - http://bmjopen.bmj.com/site/about/guidelines.xhtml

Aims and objectives

The overall aim of the present study is to assess the effects of implementing PCA throughout Region Stockholm, using a naturalistic prospective study design. This involves a number of quantitative studies which intend to understand the impact of PCA on hospitalization rates and self-reported outcomes when introduced in psychiatric care. Qualitative studies will also be conducted to explore the experiences of both patients and healthcare providers with regard to its effects and use, including the perspective of caregivers of adolescents.

Primary research question:

For patients with severe psychiatric conditions, identified as having extensive care needs and who have been assigned PCA by their healthcare providers; how will their hospitalization rates change during the following 12, 24, and 36 months with regard to the number of admissions and days in inpatient care, and the number of admissions and days in involuntary care?

Secondary research question:

1) Will hospitalization rates differ between the various patient groups who have been assigned PCA?

2) Will self-reported outcomes change following the assignment of PCA, i.e., symptoms, quality of life, and autonomy?

3) Will self-reported outcomes differ between the various patient groups who have been assigned PCA?

4) How are the health care expenditures for patients who have been assigned PCA affected, and do they differ between the various patient groups?

5) What are the experiences of patients who have been assigned PCA for at least 12 months?

6) What are the experiences of healthcare providers who have worked with PCA for at least 12 months?

7) For adolescents, what are the experiences of caregivers to patients who have been assigned PCA for at least 12 months?

8) For adolescents, will self-reported outcomes change in terms of their caregivers' perception of the patients' symptoms and quality of life?

In line with prior research, the present study hypothesizes that a) the hospitalization rates of patients will increase in terms of number of admissions to inpatient care, but b) decrease in terms of days in inpatient care, as well as number of admissions and days in involuntary care. Furthermore, self-reported outcomes are expected to demonstrate c) a decrease in symptoms, and d) increase in quality of life and autonomy. Lastly, qualitative investigations are believed to give a greater understanding of how patients, caregivers of adolescents, and healthcare providers experience the use of PCA.

Method and analysis

Setting

For peer review only

The present study is placed within the psychiatric care of Region Stockholm, which is the largest hospital sector in Sweden, serving a population of two million citizens. It is organized as nine public healthcare clinics, and four private healthcare clinics. One of these clinics constitute child and adolescent psychiatry, two concern eating disorders, and two are dedicated to substance use disorders. One additional clinic is reserved for forensic psychiatry, but because its patients have been sentenced to care, these are not eligible to receive PCA. The 13 clinics that are included consist of approximately 45 inpatient wards and about 120 outpatient units that work together as pairs (i.e., more than one outpatient unit can share the same inpatient ward). Each inpatient ward will in turn designate at least one bed for PCA. The healthcare providers consist of medical doctors and nurses with or without specialist training in psychiatry, mental health workers, counsellors, psychologists, psychotherapists, social workers, physiotherapists, occupational therapists, and students undergoing basic or advanced clinical training in the field of medicine or psychiatry. The starting date of the present study was January 1, 2022, and the planned end date is December 31, 2027.

Patients
In Region Stockholm, PCA is assigned to those patients that have a severe psychiatric condition and who are deemed by their healthcare providers to warrant great care needs. Thus, eligible patients present with a documented history of recurrent admissions to inpatient care, a large number of days in inpatient care, or other indications of future hospitalization. In order to determine eligibility, the following criteria are used by the healthcare providers:

- Ongoing contact with an outpatient unit.
- An individual care plan and crisis plan.
- At least one episode of inpatient care during the previous one-year period.
- Expected future need of inpatient care.
- Demonstrating an interest in and understanding the concept of PCA (for adolescents, this also includes their caregivers).

The inpatient wards include patients from general psychiatry (e.g., schizophrenia spectrum diagnoses, neuropsychiatric disorders, and anxiety disorders), geriatric psychiatry, affective disorders (e.g., bipolar disorder), substance use disorders, personality disorders, eating disorders, and child and adolescent psychiatry (i.e., up to 18 years of age).

Procedure
Patients who are eligible to receive PCA are invited to a scheduled meeting with their healthcare providers from the outpatient unit and inpatient ward. The purpose and practice of PCA are then discussed verbally and information is provided in writing. Should the patient accept the offer, an agreement on the use of PCA is reached collaboratively and recorded in their medical records. For adolescents, caregivers also have to agree. Typically, PCA involves up to four days of inpatient care at a time, up to three times per month, although exceptions to the rule can sometimes be made. The agreement only covers 12 months at a time, after which it can be renewed at the yearly assessments. After the meeting, the patient receives all of the details in text and a printed copy of the agreement.

Each inpatient ward has allocated at least one inpatient bed for patients assigned PCA. Should a patient with an agreement wish to be admitted, they only have to contact the inpatient ward, any time of the day, any day of the week. In case the inpatient bed is already occupied, the patient is put on a waiting list. If more immediate care is required, the patient can always be admitted as usual. All admissions are managed by a nurse with delegated

For peer review only

responsibility from the head of clinic. During admission, the patient is assessed with regard to suicidality, violence, and vital parameters, such as blood pressure and body temperature. Should there be an increased risk (e.g., suicidal ideation and physical injury), regular intake is made. Once admitted, patients are offered care according to their specific needs and individual care plan. Consultation with a medical doctor is possible, but not part of usual practice. The patient is also free to discharge at will, which is also handled by a nurse; however, should the patient warrant further stay at the inpatient ward, a medical doctor can decide if regular admittance is necessary.

Since PCA is part of regular healthcare, patients can always agree to its use but refuse to participate in the present research study. The offer to take part in a study on the effects of PCA is always made after the use of PCA first has been agreed upon, whereupon the objectives of the present study is discussed verbally and information is provided in writing. Informed consent is required and documented in the medical records. For adolescents, informed consent needs to be signed by both the adolescent and all caregivers (e.g., legal parents, custodians, or other entities responsible for the patient's care). Following this, the patient completes the self-reported outcome questionnaires that are included in the study, as reviewed below. For adolescents, this also involves the caregivers' perception of the patient's well-being. All of the self-reported outcome questionnaires are then completed at the mandatory yearly assessments, except for the Visual Analogue Scales (VAS) that are administered each month via SMS (more information provided under self-reported outcomes). This is in line with the procedures surrounding the use of PCA in Region Stockholm, where mandatory yearly assessments are made to check up on patients' health status and whether or not PCA should be continued. This is also believed to improve data collection as it does not require paper-and-pencil administration or letters.

The implementation of PCA throughout Region Stockholm is coordinated and supported by the Centre for Psychiatry Research, which is a R&D center run jointly by Karolinska Institutet and the hospital sector. The Centre for Psychiatry Research performs an assessment of the risks and needs of each outpatient unit and inpatient ward, and provides training and supervision regarding all of the PCA routines. This consists of an online course and a half-day on-site workshop, and the latter of regular meetings with all of the healthcare providers. The Centre for Psychiatry Research is also responsible for evaluating the effects of PCA and has a research steering group consisting of the authors of the current study protocol. These range from doctoral candidates to senior-level researchers with comprehensive experience of conducting both quantitative and qualitative studies in a psychiatric setting.

Study design

The present study will evaluate the effects of PCA in Region Stockholm in collaboration with the Commissioner of Healthcare. This follows a resolution made by the popularly elected Region Stockholm Assembly, which means that both the implementation and policy surrounding PCA have already been decided. Hence, according to the decision, all patients with severe psychiatric conditions who are considered by their healthcare providers to have great care needs will be offered PCA as long as the criteria mentioned above are fulfilled. Furthermore, because the primary objective of the PCA program is the provision of healthcare and not research, a more complex study design using randomization on an individual or clinic level (i.e., cluster) is not allowed due to ethical and judicial reasons. Hence, the present study is restricted to a naturalistic prospective study design. Patients are followed for 12, 24, and 36 months using their medical records to check changes in hospitalization prior to (pre) and following inclusion in the PCA program (post), similar to

For peer review only

what has been done previously.[7] For example, patients who are included in November 2021 may have hospitalization rates retrieved from the period November 2018-2020 and compared against hospitalization rates from the period November 2022-2024, depending on the availability of historical data. In terms of self-reported outcomes, patients complete the measures when being assigned PCA and at the yearly follow-up assessments, with the exception for the VAS that is administered monthly via SMS. However, in these specific cases, historical data do not exist as these measures have not been collected previously. Only patients with no previous use of PCA when they consent to take part in the present study will be analyzed, as recorded in their hospital records.

Patient and public involvement
The present study is conducted in collaboration with the Swedish Partnership for Mental Health (NSPH). Patient and public representatives are not involved in issues surrounding study design, but are informed about and actively engaged in formulating research questions, determining outcomes, and disseminating the results.

Demographic variables
Demographic variables are recorded in the patients' medical records through their social security number. These are age, gender, civil status, and children. Psychiatric disorders are entered manually by healthcare providers using the Swedish version of the International Classification of Diseases and Related Health Problems, 10th Revision (ICD-10-SE).

Hospitalization rates
Hospitalization rates are recorded in the patients' medical records when admitted to inpatient care or involuntary care (i.e., when and how long each stay lasted). This consists of number of admissions and days in inpatient care, and number of admissions and days in involuntary care. All of the healthcare providers have been trained in how to enter this information in the system, including coding schemes specifically designed for the present study in order to identify and track patients.

Self-reported outcomes
Self-reported outcome (i.e., questionnaires) are collected after the patients have been assigned PCA and consented to take part in the research. For an overview, see Table 1.

Table 1
Overview of The Self-Reported Outcomes

	Assessment				
**Self-report measure**	**Assigned PCA**	**Monthly measures**	**12 months**	**24 months**	**36 months**
**Adults**					
EQ-5D-5L	X		X	X	X
WHODAS	X		X	X	X
CGI[a]	X		X	X	X
BBQ	X		X	X	X
GSE	X		X	X	X
GAD-7	X		X	X	X
PHQ-9	X		X	X	X
VAS	X	X	X	X	X
**Adolescents**					

For peer review only

KIDSCREEN	X		X	X	X
ASSIST-Y	X		X	X	X
C-GAS[a]	X		X	X	X
SDQ[b]	X		X	X	X
RCADS[b]	X		X	X	X

WHODAS = World Health Organization Disability Assessment Schedule 2.0; CGI = Clinical Global Impression; BBQ = Brunnsviken Brief Quality of Life Scale; GSE = General Self-Efficacy Scale; GAD-7 = Generalized Anxiety Disorder – 7 Items; PHQ-9 = Patient Health Questionnaire – 9 Items; VAS = Visual Analogue Scales; ASSIST-Y = Alcohol, Smoking and Substance Involvement Screening Test; C-GAS = Children's Global Assessment Scale; SDQ = The Strengths and Difficulties Questionnaire; RCADS = Revised Children's Anxiety and Depression Scale
[a] Clinician-rating scale
[b] Also completed by caregivers

EQ-5D-5L: A five-item questionnaire on current health status on the dimensions; mobility, self-care, usual activities (e.g., family), pain/discomfort, and anxiety/depression. Each item is scored in relation to a five-step scale, e.g., "I am not anxious or depressed" (1) to "I am extremely anxious or depressed" (5). Furthermore, EQ-5D-5L also includes a VAS on current health status; "Worst imaginable health" (0) to "Best imaginable health" (100).[13]

World Health Organization Disability Assessment Schedule 2.0 (WHODAS): A 12-item questionnaire on overall physical and psychological well-being, e.g., "In the past 30 days, how much difficulty did you have in: Taking care of household responsibilities?". It is scored on five-point Likert-scale, "None" (1) to "Extreme or cannot do" (5). Three additional items also concern the number of days difficulties were present or interfered with daily living.[14]

Clinical Global Impression (CGI): A clinician-rating scale that asks healthcare providers to rate the health status of a patient at an initial assessment (referred to CGI-S); "Normal, not at all ill" (1) to "Among the most extremely ill patients" (7). The CGI is also used to determine the improvement of a patient (referred to as CGI-I); "Very much improved since the initiation of treatment" (1) to "Very much worse since the initiation of treatment" (7).[15]

Brunnsviken Brief Quality of Life Scale (BBQ): A 12-item questionnaire on the quality of life in six domains; leisure time, view of life, creativity, learning, friends and friendship, and myself as a person. Each domain is scored in accordance with satisfaction and importance, "Do not agree at all" (0) and "Agree completely" (4), which are then multiplied. The sum of the six products constitutes the total quality of life score.[16]

General Self-Efficacy Scale (GSE): A 10-item questionnaire on self-efficacy, i.e., the belief to succeed in a given situation or task, e.g., "I can always manage to solve difficult problems if I try hard enough" (Item 1). It is scored on a four-point Likert-scale, "Not at all true" (1) to "Exactly true" (4).[17]

Generalized Anxiety Disorder – 7 Items (GAD-7): A seven-item questionnaire on symptoms of anxiety and worry, e.g., "How often have they been bothered by the following over the past 2 weeks? Feeling nervous, anxious, or on the edge" (Item 1). It is scored on a four-point Likert-scale, "Not at all" (0) to "Nearly every day" (3).[18]

Patient Health Questionnaire – 9 Items (PHQ-9): A nine-item questionnaire on symptoms of depression and mood, e.g., "How often have they been bothered by the following over the

For peer review only

past 2 weeks? Little interest or pleasure in doing things?" (Item 1). It is scored on a four-point Likert-scale, "Not at all" (0) to "Nearly every day" (3).[19]

Visual Analogue Scales (VAS): Four items assessing different aspects of the patients' care needs and current health status are administered monthly via SMS. These include the following; 1) "I feel confident about receiving care when I need it" 2) "I am able to actively participate in my care", and 3) "I believe care is available when needed". These are scored on a continuum ranging from "I do not agree at all" (0) to "Totally agree" (10). Also, a fourth item concerns "How would you rate your current health status?", which is rated between "Worst possible health" (0) and "Best possible health" (10).

For adolescents and their caregivers, a separate set of self-reported outcomes will be used.

KIDSCREEN: An 11-item questionnaire on aspects related to health-related quality of life, e.g., "Thinking about the last week: have you been able to do the things that you want to do in your free time?" (Item 1). It is scored on a five-point Likert-scale, "Not at all" or "Never" (0) to "Extreme" or "Always" (4). One item is supposed to reflect the participant's overall health status, i.e., "In general, how would you say your health is?". KIDSCREEN is administered to both the patients and their caregivers.[20]

Alcohol, Smoking and Substance Involvement Screening Test (ASSIST-Y): A seven-item questionnaire on alcohol, substance, and tobacco use among adolescents. It screens for the consumption and negative effects of different compounds, similar to AUDIT and DUDIT, weighting scores differently depending on frequency and impact of their use.[21]

Children's Global Assessment Scale (C-GAS): A clinician-rating scale that asks healthcare providers to rate the health status of a patient. It is scored on a continuum ranging from "Needs constant supervision" (1-10) to "Superior functioning" (100).[22]

The Strengths and Difficulties Questionnaire (SDQ): A 25-item questionnaire on the psychological well-being of an adolescent, e.g., "Often loses temper" (Item 5). It is scored on a three-point Likert-scale, "Not true" (0) to "Certainly true" (2). SDQ is administered to both the patients and their caregivers.[23]

Revised Children's Anxiety and Depression Scale (RCADS): A 47-item questionnaire covering different symptoms of different psychiatric conditions, e.g., "I worry that something bad will happen to me" (Item 27). It is scored on a four-point Likert-scale, "Never" (0) to "Always" (3). RCADS is administered to both the patients and their caregivers.[24]

A separate study will also investigate how the use of PCA among adolescents affect their caregivers' quality of life (BBQ), functioning (Burden Assessment Scale),[25] and the perceived reception by healthcare providers (Family Involvement and Alienation Questionnaire).[26]

Sample size calculation
The present study intends to examine the impact of introducing PCA in psychiatric care in Region Stockholm. As such, only patients who are assigned PCA and have consented to take part in the research are included. An a priori sample size calculation, which is typically used to estimate an adequate sample size for subsequent recruitment, is thus not informative. However, based on previous experiences of introducing PCA in the region, it is reasonable to

For peer review only

expect about twelve patients per inpatient ward who consent to participate, which would amount to a total sample size of 564.[7] When calculating the standardized effect size possible to detect under these circumstances, based on the difference between two dependent means, and using a probability level of .05 and a power of 0.80, the result is Cohen's *d* of 0.12, which is a small, albeit clinically relevant effect for a population of patients with severe psychiatric disorders, and in line with similar findings on self-reported outcomes.[4 8 9]

Statistical analysis

Previous studies on the use of PCA have demonstrated that hospitalization rates are seldom normally distributed. In essence, few patients will use the possibility to admit themselves to inpatient care, resulting in data that is positively skewed and has a leptokurtic distribution. Investigating whether assumptions of normality are violated will therefore be essential prior to any statistical analysis, whereby suitable means of analyzing the data will be employed, that is, non-parametric tests or bootstrapping procedures. As for the self-reported outcomes, no prior evidence seems to suggest that data will not be normally distributed. Here, conditional changes over time will instead be modeled using a mixed models-approach, i.e., multilevel models. All analyses will be made according to an intent-to-treat principle, accounting for those values that have been lost via multiple imputation or maximum likelihood estimation. Likewise, all analyses will control for potential confounders, such as hospitalization rates prior to PCA and self-reported outcomes when assigned PCA, as well as demographics available in the hospital records, e.g., age, gender, civil status, children, and psychiatric disorders.

Apart from studying the results of introducing PCA, the present study will also assess its cost-effectiveness, similar to a newly published study from Denmark.[10] This includes determining the direct and total healthcare costs prior to and after being assigned PCA. Direct costs are the expenditures associated with each visit to a psychiatric emergency department, inpatient care, and involuntary care. Such expenditures are calculated from standardized price estimates provided by Region Stockholm, resulting in an approximation of how expenditures change following the implementation of PCA. Total healthcare costs are the expenditures of other types of healthcare, e.g., visits to primary care. Moreover, cost-utility analyses will also entail so-called Quality Adjusted Life Years (QALYs), which represent the improvement in terms of well-being for each incremental increase in costs by allowing patients to admit themselves, i.e., a cost-utility approach. QALYs can be derived from self-reported outcomes on the EQ-5D-5L, which is commonly used for this purpose, using population-generated preference weights which are available for a Swedish setting.[27] This will be compared to matched patients using registries. In addition, probability sensitivity analyses will be conducted to determine the errors that surrounds the cost-utility calculations made. This provides confidence ellipses at 50%, 75% and 95% that represent the uncertainty associated with each estimate.

Qualitative studies

In addition to the quantitative studies, the present study will also involve a number of qualitative investigations to further the current understanding of its use and effects, similar to previous research in the field[28 29]. First, patients who have been assigned PCA for at least 12 months will be recruited to share their perspective (n = 30). Recruitment will be purposeful to ensure heterogeneity with regard to patient groups and the extent to which PCA have been used. The aim is to explore how PCA has been perceived and what impact it might have had on both day-to-day activities and quality of life. Second, healthcare providers from different inpatient wards and professional backgrounds, having had at least 12 months of experience of

For peer review only - http://bmjopen.bmj.com/site/about/guidelines.xhtml

working with PCA, will be recruited to provide their view of its application (n = 30). The aim is to investigate their attitudes toward the method in general, the implementation of PCA in particular, and whether these have changed over time as a result of working with PCA. Third, caregivers of adolescents who have been assigned PCA for at least 12 months will be recruited to share their experiences (n = 20), both in terms of how they have perceived its effects for the adolescent and how it might have affected their own situation. All of the qualitative studies will be made using individual semi-structured interviews that are recorded digitally and transcribed ad verbum. Given the exploratory nature of this research, all analyses will be inductive and done in accordance with thematic analysis.[30] This approach is often used in social sciences as well as in health sciences and helps to understand the viewpoint of an individual in relation to a specific topic, phenomenon, or subject matter. This can be particularly useful in examining patterns and concepts in shared responses, which is helpful in generating hypotheses in a research field that lacks prior research.

Ethics and dissemination

The present study was approved in March 2021 by the Swedish Ethical Review Authority (Dnr: 2020-06498), and has been registered as a clinical trial on www.ClinicalTrials.gov (NCT04862897). All data is recorded using Region Stockholm's public healthcare's digital medical records system, TakeCare, and only exported for statistical analyses in an aggregated pseudonymized format. Moreover, participant identifiers instead of Swedish social security numbers are used to ensure anonymity, e.g., LSR64UFL. To participate in the research, informed consent has to be provided, and for adolescents, this includes caregivers (e.g., legal parents, custodians, or other entities responsible for the patient's care).

Given the novelty of the intervention and lack of research concerning PCA, the present study has the potential to make a significant contribution to the field. Apart from examining its effects with regard to hospitalization rates, self-reported outcomes will help to investigate possible benefits on symptoms, quality of life, and autonomy. In addition, qualitative studies will provide useful insights regarding its use and implications for care and everyday life, including the opinions of caregivers of adolescents. In order to successfully disseminate the results, findings will be presented in international peer-reviewed journals, at scientific conferences, and as part of two doctoral theses. In addition, an ongoing collaboration with patient representatives will ensure Patient Public Involvement and dissemination to patients and their significant others, primarily through the Swedish Partnership for Mental Health (NSPH).

Author's contributions

AR, SS, TL, NJL, MSL, EA, AB, CH, MS and LF have all been involved in the design and planning of the present study. All authors have taken part in drafting the manuscript and are also part of the steering group for researching PCA in the region.

Funding statement

The present study, its implementation, and research is funded by the Commissioner of Healthcare in Region Stockholm and Vetenskapsrådet (2021-06378).

Competing interest statement

None declared.

References

For peer review only

1. Strand M, von Hausswolff-Juhlin Y. Patient-controlled hospital admission in psychiatry: A systematic review. *Nord J Psychiatry* 2015;69(8):574-86. doi: 10.3109/08039488.2015.1025835 [published Online First: 2015/04/03]
2. Pelto-Piri V, Engström K, Engström I. Paternalism, autonomy and reciprocity: ethical perspectives in encounters with patients in psychiatric in-patient care. *BMC medical ethics* 2013;14(1):1-8.
3. Koekkoek B, van der Snoek R, Oosterwijk K, et al. Preventive psychiatric admission for patients with borderline personality disorder: a pilot study. *Perspect Psychiatr Care* 2010;46(2):127-34. doi: 10.1111/j.1744-6163.2010.00248.x [published Online First: 2010/04/10]
4. Moljord IEO, Lara-Cabrera ML, Salvesen O, et al. Twelve months effect of self-referral to inpatient treatment on patient activation, recovery, symptoms and functioning: A randomized controlled study. *Patient Educ Couns* 2017;100(6):1144-52. doi: 10.1016/j.pec.2017.01.008 [published Online First: 2017/01/18]
5. Moljord IE, Helland-Hansen KA, Salvesen O, et al. Short time effect of a self-referral to inpatient treatment for patients with severe mental disorders: a randomized controlled trial. *BMC Health Serv Res* 2016;16(1):513. doi: 10.1186/s12913-016-1712-z [published Online First: 2016/09/24]
6. Thomsen CT, Benros ME, Maltesen T, et al. Patient-controlled hospital admission for patients with severe mental disorders: a nationwide prospective multicentre study. *Acta Psychiatr Scand* 2018;137(4):355-63. doi: 10.1111/acps.12868 [published Online First: 2018/03/06]
7. Skott M, Durbeej N, Smitmanis-Lyle M, et al. Patient-controlled admissions to inpatient care: A twelve-month naturalistic study of patients with schizophrenia spectrum diagnoses and the effects on admissions to and days in inpatient care. *BMC Health Serv Res* 2021;21(1):598. doi: 10.1186/s12913-021-06617-8 [published Online First: 2021/06/25]
8. Westling S, Daukantaite D, Liljedahl SI, et al. Effect of Brief Admission to Hospital by Self-referral for Individuals Who Self-harm and Are at Risk of Suicide: A Randomized Clinical Trial. *JAMA Netw Open* 2019;2(6):e195463. doi: 10.1001/jamanetworkopen.2019.5463 [published Online First: 2019/06/08]
9. Strand M, Bulik CM, Gustafsson SA, et al. Self-admission to inpatient treatment in anorexia nervosa: Impact on healthcare utilization, eating disorder morbidity, and quality of life. *Int J Eat Disord* 2020 doi: 10.1002/eat.23346 [published Online First: 2020/07/16]
10. Paaske LS, Sopina L, Olsen KR, et al. The impact of patient-controlled hospital admissions among patients with severe mental disorders on health care cost: A nationwide register-based cohort study using quasi-experimental design. *J Psychiatr Res* 2021;144:331-37. doi: 10.1016/j.jpsychires.2021.10.032 [published Online First: 2021/11/06]
11. Olso TM, Gudde CB, Moljord IE, et al. More than just a bed: mental health service users' experiences of self-referral admission. *Int J Ment Health Syst* 2016;10:11. doi: 10.1186/s13033-016-0045-y [published Online First: 2016/02/27]
12. Eckerström J, Flyckt L, Carlborg A, et al. Brief admission for patients with emotional instability and self-harm: A qualitative analysis of patients' experiences during crisis. *International Journal of Mental Health Nursing* 2020 doi: 10.1111/inm.12736
13. Herdman M, Gudex C, Lloyd A, et al. Development and preliminary testing of the new five-level version of EQ-5D (EQ-5D-5L). *Quality of life research* 2011;20(10):1727-36.

For peer review only

14. Üstün TB, Kostanjsek N, Chatterji S, et al. Measuring health and disability: Manual for WHO disability assessment schedule WHODAS 2.0: World Health Organization 2010.
15. Busner J, Targum SD. The Clinical Global Impressions Scale: Applying a research tool in clinical practice. *Psychiatry (Edgmont)* 2007;4(7):28-37.
16. Lindner P, Frykheden O, Forsstrom D, et al. The Brunnsviken Brief Quality of Life Scale (BBQ): Development and Psychometric Evaluation. *Cogn Behav Ther* 2016;45(3):182-95. doi: 10.1080/16506073.2016.1143526 [published Online First: 2016/02/18]
17. Sherer M, Maddux JE, Mercandante B, et al. The self-efficacy scale: Construction and validation. *Psychological reports* 1982;51(2):663-71.
18. Spitzer RL, Kroenke K, Williams JB, et al. A brief measure for assessing generalized anxiety disorder: the GAD-7. *Archives of internal medicine* 2006;166(10):1092-97.
19. Kroenke K, Spitzer RL, Williams JB. The PHQ-9: validity of a brief depression severity measure. *Journal of general internal medicine* 2001;16(9):606-13.
20. Ravens-Sieberer U, Gosch A, Rajmil L, et al. KIDSCREEN-52 quality-of-life measure for children and adolescents. *Expert review of pharmacoeconomics & outcomes research* 2005;5(3):353-64.
21. Humeniuk R, Holmwood C, Beshara M, et al. ASSIST-Y V1. 0: First-Stage Development of the WHO Alcohol, Smoking and Substance Involvement Screening Test (ASSIST) and linked brief intervention for young people. *Journal of Child & Adolescent Substance Abuse* 2016;25(4):384-90.
22. Shaffer D, Gould MS, Brasic J, et al. A children's global assessment scale (CGAS). *Archives of General psychiatry* 1983;40(11):1228-31.
23. Goodman R. The Strengths and Difficulties Questionnaire: a research note. *Journal of child psychology and psychiatry* 1997;38(5):581-86.
24. Chorpita BF, Yim L, Moffitt C, et al. Assessment of symptoms of DSM-IV anxiety and depression in children: A revised child anxiety and depression scale. *Behaviour research and therapy* 2000;38(8):835-55.
25. Ewertzon M, Lützén K, Svensson E, et al. Developing the concept of family involvement and the alienation questionnaire in the context of psychiatric care. *International Journal of Mental Health Nursing* 2008;17(6):439-49.
26. Reinhard SC, Gubman GD, Horwitz AV, et al. Burden assessment scale for families of the seriously mentally ill. *Evaluation and program planning* 1994;17(3):261-69.
27. Burström K, Sun S, Gerdtham U-G, et al. Swedish experience-based value sets for EQ-5D health states. *Quality of Life Research* 2014;23(2):431-42.
28. Ellegaard T, Bliksted V, Lomborg K, et al. Use of patient-controlled psychiatric hospital admissions: patients' perspective. *Nord J Psychiatry* 2017;71(5):370-77. doi: 10.1080/08039488.2017.1302505 [published Online First: 2017/03/23]
29. Ellegaard T, Mehlsen M, Lomborg K, et al. Use of patient-controlled psychiatric hospital admissions: mental health professionals' perspective. *Nord J Psychiatry* 2017;71(5):362-69. doi: 10.1080/08039488.2017.1301548 [published Online First: 2017/03/21]
30. Braun V, Clarke V. Using thematic analysis in psychology. *Qualitative Research in Psychology* 2006;3(2):77-101. doi: 10.1191/1478088706qp063oa

For peer review only
